# Attention-deficit hyperactivity disorder (ADHD) and glial integrity: S100B, cytokines and kynurenine metabolism - effects of medication

**DOI:** 10.1186/1744-9081-6-29

**Published:** 2010-05-28

**Authors:** Robert D Oades, Maria R Dauvermann, Benno G Schimmelmann, Markus J Schwarz, Aye-Mu Myint

**Affiliations:** 1 Clinic for Child and Adolescent Psychiatry and Psychotherapy, University of Duisburg-Essen, 45147 Essen Germany; 2 Laboratory for Psychoneuroimmunology, Ludwig Maximillian's University Psychiatric Hospital, 8036 Munich, Germany; 3 Child and Adolescent Psychiatry, University of Bern, Effingerstr. 12, 3011 Bern, Switzerland

## Abstract

**Background:**

Children with attention-deficit/hyperactivity disorder (ADHD) show a marked temporal variability in their display of symptoms and neuropsychological performance. This could be explained in terms of an impaired glial supply of energy to support neuronal activity.

**Method:**

We pursued one test of the idea with measures of a neurotrophin reflecting glial integrity (S100B) and the influences of 8 cytokines on the metabolism of amino-acids, and of tryptophan/kynurenine to neuroprotective or potentially toxic products that could modulate glial function. Serum samples from 21 medication-naïve children with ADHD, 21 typically-developing controls, 14 medicated children with ADHD and 7 healthy siblings were analysed in this preliminary exploration of group differences and associations.

**Results:**

There were no marked group differences in levels of S100B, no major imbalance in the ratios of pro- to anti-inflammatory interleukins nor in the metabolism of kynurenine to toxic metabolites in ADHD. However, four trends are described that may be worthy of closer examination in a more extensive study. First, S100B levels tended to be lower in ADHD children that did not show oppositional/conduct problems. Second, in medicated children raised interleukin levels showed a trend to normalisation. Third, while across all children the sensitivity to allergy reflected increased levels of IL-16 and IL-10, the latter showed a significant inverse relationship to measures of S100B in the ADHD group. Fourthly, against expectations healthy controls tended to show higher levels of toxic 3-hydroxykynurenine (3 HK) than those with ADHD.

**Conclusions:**

Thus, there were no clear signs (S100B) that the glial functions were compromised in ADHD. However, other markers of glial function require examination. Nonetheless there is preliminary evidence that a minor imbalance of the immunological system was improved on medication. Finally, if lower levels of the potentially toxic 3 HK in ADHD children were confirmed this could reflect a reduction of normal pruning processes in the brain that would be consistent with delayed maturation (supported here by associations with amino-acid metabolism) and a reduced metabolic source of energy.

## Background

Childhood attention-deficit/hyperactivity disorder (ADHD) is characterised by poor attention-related abilities, restless activity along with cognitive and behavioural impulsivity. All 18 features of the diagnosis are prefaced by the word "often" in the DSM-IV. Such variability is also typical of laboratory measures of the children's abilities, and is well illustrated by response times (RTs) in neuropsychological tests for all subgroups [1-3]. Thus, a core feature of childhood ADHD is the variability of the nature and timing of behavioural responses. This variability, typically measured on RT tasks, is a good candidate for an endophenotype of the disorder [4,5]. A seminal hypothesis was proposed to explain a potential biological basis for this phenomenon [6]. In essence, these authors suggested that the ability to sustain the rapid neuronal firing underlying responsivity requires the maintenance of the supply of energy (the lactate shuttle) from the supporting glial cells (the astrocytes). A deficient energy supply could result in the disruption of responsivity seen as variable on/off responding in ADHD.

This pilot study examines levels of the cytokine-related neurotrophin S100B as a putative biomarker of the integrity of glial function, hypothesising that altered levels could reflect dysfunction and an impaired energy supply for neuronal activity. Serum S100B levels correlate strongly with CSF measures [7] and they largely, but not exclusively, derive from astrocytes and oligodendrocytes [8]. S100B is involved in the regulation of glutamate and calcium uptake, neuronal plasticity, energy metabolism and in various neurodevelopmental processes [9], especially those involving the neurotrophic role of serotonin (5-HT: [10]). Over production of S100B (micromolar levels) can be toxic and follow brain-damage resulting from stroke and ischemia [11] and accompany severe mental disorders such as schizophrenia, bipolar disorder, depression, Alzheimer's disease, Down's syndrome, phenylketonuria and cerebral palsy [12-15]. In some of these disorders variability features strongly [6,16].

However, in cell culture prolonged glucose deprivation can down-regulate S100B mRNA expression and eventually lead to reduced S100B release [17]. A regional reduction of S100B levels has also been reported in rats subjected to prenatal stress [18]. In brain slices reduced S100B secretion has been associated with high levels of potassium ions [19]. Indeed, high levels of potassium are a prediction of Russell's hypothesis on deficient energy supplies in ADHD [6]. As ADHD is a disorder with qualitative and quantitative differences from severe mental illness such as the psychoses, we hypothesised that levels of the cytokine may be reduced reflecting inefficient control of the energy supply to active neuronal pathways.

Markers of one of the potential causes of glial dysfunction were also sought. These markers reflect the nature of tryptophan metabolism. From 80 to 95% of brain levels of l-tryptophan are metabolised in the kynurenine pathway with the remainder contributing to the synthesis of 5-HT [20]. Activity in these two pathways merits attention for two reasons. First, there is good reason to believe that levels of 5-HT activity in ADHD are anomalous [21-23]. Second, catabolic products of kynurenine can be neuroprotective (Kynurenic acid, KA, a glutamate antagonist) or potentially neurotoxic (3-OH-Kynurenine, 3HK, a glutamate agonist:[24]), where 3HK is a precursor to the production of toxic quinolinic acid. Alternatively this metabolic pathway could be considered a step in the production of nicotine adenine dinucleotide (NAD) as a source of energy for neuro-glial networks. Clearly unusual levels of these substances could be indicative of factors influencing anomalous glial function.

Finally, an important determinant of the production of glial kynurenine and its metabolites is the activity of the indoleamine and tryptophan 2, 3-deoxygenases (IDO and TDO). The activity of these enzymes is modulated in part by the balance between pro- and anti-inflammatory interleukins. An imbalance would also provide evidence for or against anomalous kynurenine metabolism and glial function. Altered ratios of the interleukins and kynurenine metabolism have indeed been described for depression and bipolar illness [25-27].

The proinflammatory cytokines suitable for measurement include the interleukins IL-1β, IL-2, IL-6, tumour necrosis factor-α (TNF-α) and interferon-gamma (IFN-γ) for contrast with the antiinflammatory cytokines IL-10 and IL-13 [28]. Also included in the analyses were IL-4 (where levels proved to be below detection limits) and IL-16 that stimulates proinflammatory cytokine production but has been reported also to have antiinflammatory properties in different disorders. We are aware of only one early report on interleukin levels in childhood disorders (IL-2, IFN-γ, IL-4, IL-5, IL-10, and TNF-α). In CSF samples from patients with childhood onset of obsessive compulsive disorder, ADHD and schizophrenia there was a skew towards type-1 interleukins in the former, a preponderance of type-2 in the latter disorders with values from the ADHD patients being intermediate [29]. However, there are suggestions from genetics' studies that alterations in the interleukin balance could contribute to ADHD. The gene for the IL-1 receptor (IL-1Ra) can have 2 or 4 repeats. An initial study of 86 children with ADHD and their parents showed that transmission of the former was associated weakly with a decreased risk, and of the latter with an increased risk of transmission [30]. But, a second study of 179 families could not replicate this result [31]. A further study of IL-6 reported a marginal preponderance of the C-allele in ADHD and that an association was noted with the DRD2 Taq1A allele in those children who responded to methylphenidate treatment [32]. A recent genome wide scan of 958 ADHD child-parent trios reported that 2 single nucleotide polymorphisms (SNPs) in the IL-16 gene were associated with the inattentive phenotype, and a second analysis of 930 patients described a nominal association for IL-3 (NFIL3: C allele) with an earlier onset of the disorder [33,34]. The results of these interleukin studies are equivocal, but provide indications of a disturbance that merits closer examination.

Lastly, our analysis included a number of amino acids not only as a control for the tryptophan measures, but because branched-chain amino acids can provide an alternative energy supply to neurons. Amino acids such as leucine, isoleucine and valine can be broken down in glial cultures to metabolites that can enter the tricarboxlic acid cycle directly or even produce lactate itself [35-37].

In summary, we report on a pilot study of serum samples from medication-naïve children with and without ADHD, and a group taking psychostimulants for 1) S100B, 2) tryptophan metabolites, 3) 17 amino-acids, and 4) a set of pro- and antiinflammatory interleukins as potential indicators of the integrity of glial cell function (hypothesis 1: decreased in ADHD), of the presence of toxic over-protective features of amino-acid metabolism (hypothesis 2: in ADHD) and the immunological activity influencing metabolism (hypothesis 3: an imbalance). A high variability of RT (coefficient of variance) for children with ADHD was established on a continuous performance task (CPT) prior to entry to the study.

## Methods

### Subjects

Participants included 21 medication-naïve children referred consecutively to the Clinic for Child and Adolescent Psychiatry in Essen who were given a DSM-IV diagnosis of ADHD combined type. A second older group of 14 children for whom the same diagnosis had been made 4 years previously and were receiving medication were also recruited (ADHDmed). One was receiving atomoxetine, one Ritalin and the others a retard formulation (Concerta or Medikinet, 30-40 mg). A control group (CON) of 21 typically developing children for the ADHD group was recruited by advertisement. An attempt to recruit controls for the ADHDmed group from their healthy siblings resulted in only 7 participants (SIBS). The gender, age, IQ, and body-mass index (BMI) of the children, and the socio-economic status of the father are shown in table 1. In accord with the Declaration of Helsinki, the protocol for the study was approved by the ethics committee of the University Medical Faculty: parents and children received verbal and written information and gave written consent to the procedures.

**Table 1:** Characteristics of the subject groups (means with standard deviations, SD, and range of values) and the serum concentrations of S100B (pg/ml)

	N	Gender m/f	Age years	IQ	BMI	Allergy severity	SES F-c	S100B (pg/ml)
ADHD	21	14/7	8.84	95.8	16.5	0.86	4.3	93.89
SD			(1.4)	(10.7)	(2.4)	(1.2)	(2.2)^#^	(17.63)^#^
Range			6.6-11.7	82-124	13.9-24.8	0-3 Np8 Nc1	1-7	70-152
CON	21	20/1	11.0	114.1	17.9	0.76	3.5	97.21
SD			(1.5)	(14.4)	(3.1)	(0.9)	(2.1)	(31.38)^#^
Range			7.7-13.4	92-141	13.5-24.9	0-3 Np11 Nc 1	1-7	55-156
ADHDmed	14	12/2	12.6	106.7	20.5	0.29	4.1	84.08
SD			(2.1)	(12.4)	(4.6)	(0.7)	(1.9)	(26.67)
Range			7.9-15.5	92-134	14.2-32	0-2 Np2 Nc 0	2-6	35-127
SIBS	7	4/3	11.7	108.1	18.5	0	4.4	96.86
SD			(2.1)	(14.0)	(2.5)	(0)	(2.0)	(34.01)
Range			9.0-14.4	87-134	15.6-23.1	0 Np0 Nc0	2-6	59-153

ADHD, children with attention deficit hyperactivity disorder (combined type); CON, healthy controls; ADHDmed, replication group of children with ADHD (medicated), SIBS, siblings without medication; BMI, body-mass index; SES, current socio-economic status of the father (F-c: scale 1-7 for professions:[100]), # N = 19. Allergy severity refers to past and current experience (scale 0-3) affecting Np individuals in the past (>12 months) or Nc individuals currently. There were more females in the ADHD and SIB groups (p < 0.05). The ADHD group was younger and had a lower IQ than the other 3 groups (Scheffe p < 0.007) but did not differ significantly on BMI, allergy or SES.

S100B levels omit as outlier values one CON (248 pg/ml) and one ADHDmed measure (385 pg/ml) which were > 3 SD above the mean.

A research diagnosis was made using a semi-structured parent interview (Parental Account of Children's Symptoms/PACS:[38,39]). Information was collected from the parent about the child's behaviour relating to 4 domains: mood disorder, ADHD/hyperkinetic disorder, disruptive behaviour and additional problems. Parents describe their child's behaviour in unstructured (e.g. playing alone), semi-structured (e.g. meal times) and structured (e.g. homework) daily life situations. The severity and frequency of the behaviour are rated according to previously defined criteria. The replies are coded on a 4-point scale for the previous week and the previous year (in an unmedicated state) on the basis of formal training and written definitions. A standardized diagnostic algorithm based on the DSM-IV criteria relating to symptoms, age of onset, situational pervasiveness and clinical impairment was applied. Previous studies have shown high inter-rater reliability with correlations between .76 and .96 [39]. Symptoms were also rated with the long forms of the Conners parent and teacher ratings scales (CPRS-R: L, CTRS-R: L, scales with 80 and 59 items, respectively: [40]). Five subscales were considered for analysis: opposition, anxiety, inattention, hyperactive-impulsive and the total overall ratings. The extent of comorbid internalizing and externalizing problems was represented by the number of children scoring T > 65 on anxiety/oppositional scales of either the CTRS or CPRS: 9/14 (ADHD), 6/2 (CON), 6/9 (ADHDmed) and 3/1 (SIBS). Exclusion criteria included a history of encephalitis, autism, epilepsy, Tourette syndrome, bipolar disorder, IQ < 80, brain damage and any genetic or medical condition associated with externalizing behaviours that might mimic ADHD.

In addition, the nature of any allergy, current or past and its severity (0-3) was also recorded. The IQ was assessed with the standardized version of the CFT-20-R [41] or for subjects younger than 9 years the Kaufman assessment battery for children [42]. The high variability of RT (coefficient of variance) and performance (errors) for children with ADHD was established on a CPT and constituted a criterion for entry into the study. The association of these results with the biochemical measures are the subject of an associated report [43].

### Biochemical analyses

Fasting venous blood (20 ml) was withdrawn in the morning (08:00-09:00), the serum separated and stored at -80°C for later analysis by technicians blind to the source of the sample [44]. S100B concentrations were measured in the serum by an immuno-luminometric assay that detects the heterodimer S100 A1B and the homodimer S100 BB (Elecsys S100™, Roche Diagnostics, Switzerland). The lowest detectable concentration of S100B was 0.02 μg/L. Intra- and inter-assay coefficients of variation were < 5% and < 8%, respectively.

The cytokines IFN-γ, IL-1β, IL-2, IL-3 IL-6, IL-10, IL-13, IL-16, LIF, and TNF-α were analysed in the serum using ELISA test kits from R&D systems as described in the manufacturer's instruction. For determination of IL-1β, IL-6, IL-10 and TNF-α we used high sensitive assays. All samples were below the detection limit for leukaemia inhibitory factor (LIF: limit: 7.8 pg/ml), and IL-3 (limit: 15.6 pg/ml), and for 40/63 samples IL-1β (detection limit: 0.063 pg/ml) was not detected. These samples were excluded from further calculation.

For amino acid determination the AccQ Tag method was used (Waters Corporation, Milford, MA, USA). This analysis concerned the 5 tryptophan-competing amino acids (isoleucine, leucine, phenylalanine, tyrosine and valine) and 12 other amino-acids (alanine, arginine, aspartate, glutamate, cystine, glycine, histidine, lysine, methionine, proline, serine and threonine). Briefly, after pre-column derivatization using 6-aminoquinolyl-N-hydroxy-succinimidyl carbamate, the samples were injected to a HPLC system consisting of a 2695 separations module connected to a 2475 fluorescence detector (Waters). The gradient used the AccQ Tag eluent, acetonitrile, and HPLC grade water with a flow rate of 1.0 ml/min at a temperature for the AccQ Tag column (3.9 × 150 mm) of 37°C. The detection wave lengths were λex = 250 nm and λem = 395 nm.

For tryptophan, kynurenine and their metabolites the analytes were extracted from samples and calibrators/controls using Waters Oasis MCX 1 cc (30 mg) extraction cartridges. The eluent was evaporated to dryness under nitrogen and reconstituted with 150 μl 0.1 M PBS. Reconstituted samples/calibrators/controls were transferred to microinjection vials and the analyses run on a Waters 2695 chromatograph connected to a Waters Model 2487 dual-l UV detector and a 2475 fluorescence detector. For determination of 5-HIAA, kynurenine, KA and 3HK, 100 μl of the samples were loaded onto a 250 mm × 4 mm Supersphere 60 RP-select B, C8 column (Merck, Darmstadt, Germany). Due to the relatively higher concentration, a second 10 μl injection was performed for the determination of tryptophan. The gradient elution used a mobile phase consisting of a mixture of 0.05 M sodium acetate (solvent A: pH 4.80; solvent B: pH 3.65), acetonitrile (solvent C), and methanol (solvent D). The flow rate was 0.80 ml/min with the column temperature set to 35.0°C, while the samples were cooled to 4.0°C. Tryptophan (lex: 300 nm; lem: 350 nm) and 5-HIAA (lex: 300 nm; lem: 340 nm) were measured by fluorescence detection; kynurenine (365 nm), KA (330 nm), and 3-HK (365 nm) were measured by UV detection. Run times after injection until detection of the compounds were about 20.4 min for tryptophan, 13.4 min for kynurenine, 22.5 min for KA, and 7.0 min for 3HK. Data were processed using EMPOWER for Windows 2000 software (Waters). The concentrations were established through comparison of peak heights of the single analytes with the peak heights of the respective calibration curves.

Three indices of activity in the tryptophan metabolic path were calculated from the above serum analyses. (1) The *tryptophan availability index*, 100 × tryptophan/sum of the competing amino acids; (2) The *tryptophan breakdown index *that reflects the sum of the activities of the TDO and ID enzymes, kynurenine/tryptophan, and (3a) the *neuroprotective index*, KA/3HK. It may be noted that neuroprotection would be increased with a larger index value. This third index differs from that used by Myint et al. [44] who calculated the ratio of 1000 × KA/kynurenine, which will be referred to here as index 3b that represents the turnover rate within the neuroprotective pathway and does not refer directly to the production of toxic metabolites.

### Data analysis

The groups were compared on demographic data and behavioural ratings (questionnaire and biochemistry) with the use of ANOVA for continuous variables and the t-test for other variables. They were compared initially after controlling for age, BMI, gender and allergy. The covariates in the final analysis are noted in the results section, and are usually restricted to age/gender as the significant correlates. (Age proved to be a good proxy for BMI in the absence of overweight cases.) Homogeneity of variance was usually evident where the larger subject groups were examined (Levene's test). The analyses consider separately S100B, the interleukins, the amino aids and kynurenine metabolites. Pearson correlations between biochemical measures were computed and, being of an exploratory nature, are reported uncorrected. Nonetheless, *P *values are 2 tailed and unless otherwise stated partial correlations that controlled for age or gender are cited. The significance of the reported tests at α = 5% can be assessed against a rough false discovery rate (FDR) criterion of p ≤ 0.02. Values up to α = 10% (FDR: p ≤ 0.05) are noted as trends or tendencies.

## Results

### S100B

Group characteristics and the mean serum concentrations of S100B are given in table 1. Overall there were no significant correlations of S100B levels with age, BMI, IQ, allergy severity or SES. S100B levels were marginally lower in the ADHD group but did not differ significantly from the CON group even after controlling for the variance due to gender, age and BMI. The slightly lower S100B levels in the medicated group did not differ significantly from the others. Contrasting all ADHD children with those without the diagnosis showed no differences in the ancova (89.9 vs. 97.1 pg/ml: F(1,54) = 1.49, p = 0.23, η^2 ^= 0.03).

We then examined whether the severity or nature of the symptoms influenced levels of S100B. Using the norm T-values for the sub-scales of the Conners' ratings to contrast ADHD- and control children with high (T ≥ 65, n = 16) and low (T ≤ 50, n = 12) total DSM or inattentive DSM CPRS ratings showed there were no differences in the levels of S100B. However, across all children the CPRS ratings of anxiety (not inattention or opposition) showed a weak bivariate tendency to relate to S100B levels (n = 56, r = +0.23, p < 0.09). Indeed, on a closer examination the presence of internalizing symptoms contributed to the relationship of decreasing S100B levels with increasing DSM total symptom ratings in the ADHD and the combined ADHD groups. In both instances the partial correlations for anxiety controlling for ratings of oppositionality approached significance (df 15/27, r = -0.54/-0.43, p = 0.027/0.020). A similar relationship was not evident for other ratings (e.g. inattention) or for the controls.

In summary, independent of age, BMI and gender there were no group differences in the level of serum S100B between those with and without a diagnosis of ADHD. However, S100B levels were lower in those children expressing higher levels of non-externalizing symptoms (e.g. mood, anxiety).

### Tryptophan metabolism

Comparison of the measures of tryptophan and 5 competing amino acids in 4 subject groups, showed a trend for higher levels of tryptophan alone in the ADHD subjects independent of medication (table 2: F(3,55) = 2.56, p = 0.06, covariates age and gender). This was confirmed in the comparison of combined ADHD vs. combined control groups (F(1,57) = 5.36, p = 0.02, η^2 ^= 0.09, power 0.62: covariates age and gender). Controlling for BMI or allergy marginally attenuated the significance (F = 3.3). Ancovas (age, gender) for tryptophan availability and breakdown confirmed higher and lower levels (respectively) in the combined ADHD than in the control groups (F(1,58) = 2.97/4.93, p = 0.09/0.03, η^2 ^= 0.05/0.08, power 0.40/0.59, respectively: table 2). Levels of the 5 competing amino acids did not differ between groups.

**Table 2:** Mean levels of tryptophan, metabolites and indices of the relative activity at 3 metabolic stages in 4 subject groups (SD in parentheses)

Group N	ADHD 20/21	CON 19/21	ADHDmed 14	SIBs 7
Tryptophan * μg/ml	11.82 *(2.16)*	10.78 *(2.23)*	12.49 *(2.69)*	11.84 *(2.55)*
Availability * Index	12.75 *(3.49)*	11.56 *(2.14)*	13.51 *(1.72)*	13.10 *(0.60)*
Kynurenine *ng/ml*	629.5 *(98.5)*	634.5 *(132.3)*	629.9 *(83.3)*	628.0 *(85.1)*
Breakdown^#^ Index	0.054 *(0.009)*	0.060 *(0.012)*	0.051 *(0.006)*	0.054 *(0.009)*
Kynurenic acid *ng/ml*	8.06 *(2.08)*	8.42 *(3.25)*	9.08 *(2.17)*	8.51 *(2.50)*
3OH-Kynurenine ^#^ *ng/ml*	18.41 *(4.43)*	20.69 *(5.05)*	19.34 *(3.66)*	19.40 *(3.65)*
Neuroprotection Index A (KA/3HK)	0.457 *(0.139)*	0.414 *(0.141)*	0.469 *(0.071)*	0.437 *(0.085)*
Neuroprotection Index B (TR)	12.93 *(3.038)*	13.52 *(4.961)*	14.34 *(2.624)*	13.57 *(3.772)*
5-HIAA ng/ml	12.08 *(3.84)*	11.84 *(5.31)*	13.97 *(6.61)*	13.79 *(5.93)*

Combined ADHD groups vs. controls * p < 0.02, ^# ^p < .08 where kynurenine levels related to 3HK in controls (r = +0.85, p < 0.000), and not in the ADHD group (p = 0.35).

Even though levels of kynurenine rose with increasing age (n63, r = +0.30, p < 0.02) it is notable that the levels of metabolites in the two different pathways for tryptophan breakdown (kynurenine and 5-HIAA) did not differ between groups with or without controlling for age, gender or BMI. However, the tendency for lower 3HK levels in the ADHD groups than in the controls (F(1,58) = 3.15, p = 0.08) merited closer attention: the same trend was evident in the neuroprotection 3a index that contrasts the KA/3HK pathways (F = 1.86; but not index 3b that reflects kynurenine/KA turnover, F = 0.03). Partial correlations (accounting for age and BMI) showed, as expected, a strong relationship for tryptophan with kynurenine (but not 5-HIAA) in ADHD, ADHDmed and control groups (r = +0.51-0.83, p = 0.02-0.001). But, in contrast to the relationships between kynurenine and the neuroprotective KA in all groups (r = +0.41-0.73, p = 0.09-0.007), the relationship of kynurenine to the toxic metabolite 3HK was marked in the controls (r = +0.85, p < 0.000), and absent in the ADHD group (p = 0.35). Medication partially restored the association in ADHDmed children (p = 0.01).

In summary, there was a tendency for ADHD children to show higher levels of tryptophan and tryptophan availability than those without ADHD. While associations of tryptophan levels with the 5-HT metabolite were not evident in any group, kynurenine breakdown was biased towards the potentially toxic 3HK in the control group alone (cf. hypothesis 2).

### Other amino-acids

**Figure 1:**
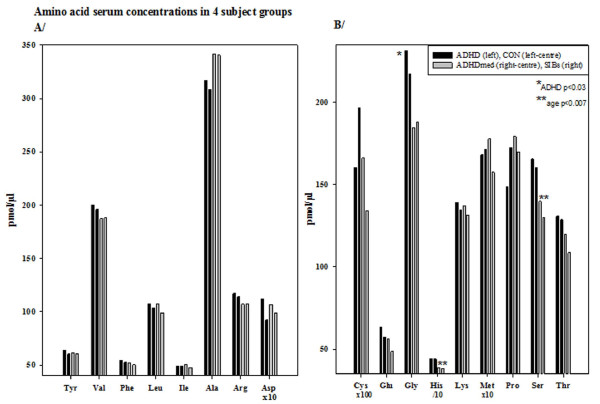
**Mean serum concentrations (pmol/μl) of 17 amino acids for 4 groups of children (ADHD left and Controls right black column; ADHDmed left and Sibs right grey columns)**. Note the different scales in figure A (left) and B (right).

Most of the 17 amino-acids studied showed non-significantly higher concentrations in the ADHD than the control groups: the exceptions with lower values were isoleucine, cystine, methionine and proline (figure 1). Concentrations decreased significantly with age (ADHD and CON vs. ADHDmed and SIBS) for histidine and serine (F(3,56) = 4.4-6.1, p = 0.001-0.007, η^2 ^= 0.19-0.25): significance levels were reduced with age as a covariate. Only glycine showed elevated levels in the ADHD group compared to each of the other groups (F(3,56) = 3.29, p = 0.028, η^2 ^= 0.15), although, noting the medium effect size, phenylalanine showed a descriptively similar trend with age as covariate (F(3,55) = 2.27, p = 0.09, η^2 ^= 0.11).

We explored bivariate correlations of the levels of amino-acids with cytokines in ADHD and control groups. The following two patterns emerged with partial correlations accounting for age. *First*, in controls S100B levels correlated positively with those for valine, leucine and isoleucine (r = +0.5, p < 0.04), methionine and threonine (r = +0.6, p < 0.02). But, in the ADHD group there was only a minor negative relationship with proline (r = -0.5, p = 0.05: note that here proline was positively correlated with IL-10, r = +0.6, p = 0.025). *Second*, in controls there were negative relationships for the antiinflammatory IL-13 with arginine (r = -0.70, p = 0.017) and the proinflammatory IL-6 with cystine (r = -0.48, p = 0.03). In contrast, the only associations for the ADHD group were that proinflammatory TNF-α levels tended to be positively related to those for alanine (r = +0.55, p = 0.029) and glutamate (r = +0.5, p = 0.05).

Exploring the correlations for amino-acid levels with kynurenine metabolism strikingly different patterns for each subject group emerged. **(1) **CON: 6 amino-acids correlated strongly and exclusively with kynurenine and 3HK: (*Kynurenine: *Val, Phe, Leu, Ile, Lys r = +0.6-0.7, p = 0.001, Met r = +0.45, p = 0.035: *3HK: *r = + 0.49-0.59, p = 0.024-0.005). **(2) **ADHD: correlations were restricted entirely to the neuroprotective index for Thr and Met (r = +0.47/0.57, p = 0.04/0.01). **(3) **ADHDmed: 12 amino-acids correlated positively with kynurenine and 10 correlated negatively with the tryptophan breakdown index and with no other item: (*Kynurenine: *Val, Phe, Leu, Ile, Arg, Lys, Met, r = +0.61-0.71, p = 0.02-0.005; Ala, Glu, Gly, Ser, Thr, r = +0.57-0.59, p = 0.025-0.04: *Try-breakdown index: *Val, Phe, Leu, Ile, Ala, Met, r = -0.67 to -0.79, p = 0.001-0.008; Tyr, Arg, Glu, Lys, r = -0.5 to 0.66, p = 0.07-0.01).

In summary, amino-acid concentrations were related differentially with cytokine levels and kynurenine metabolism in each subject group. *First *S100B levels related to some amino-acids in control but not ADHD children. *Second*, a few amino-acids were related to a proinflammatory cytokine in the ADHD group in contrast to the negative relationship seen in the controls. Striking is that many amino-acids related to kynurenine and its toxic metabolite 3HK in controls supporting the evidence (above) for marked catabolic activity in healthy children. This was not evident in the ADHD group. Medication was associated first with a reinstatement of amino acid associations with kynurenine levels, and secondly decreasing amino acid levels were inversely related to the tryptophan breakdown index.

### The cytokines

Six of the 8 interleukins measured showed non-significantly higher levels in the ADHD than control groups: only the small number of proinflammatory IL-1β measures was lower in ADHD children. TNF-α levels were similar in ADHD and CON groups (table 3). All these trends were reversed in the ADHDmed group. (Sibling data are shown for completeness, but there were too few data for analysis.) The medication effect approached significance for IFN-γ (F(1,24) = 3.43, p = 0.07, η^2 ^= 0.13) and for IL-13 (F(1,20) = 3.73, p = 0.07, η^2 ^= 0.16) after controlling for age and gender with quite strong effect sizes in both comparisons.

**Table 3:** Mean levels of cytokines (pg/ml, with standard deviations [SD]) for four subject groups

A Group	ADHD *(SD)*	CON *(SD)*	ADHDmed *(SD)*	SIBS *(SD)*	
**IL-1β**	0.23	0.35	0.62	1.25	F(2,17) = 1.30, p = 0.29.
	*0.30*	*0.30*	*0.67*	*1.39*	
(N):	(7):	(7):	(7):	(2):	
**IL-2**	7.47	6.58	6.96	6.15	F(2,40) = 0.16, p = 0.86
	*3.58*	*2.96*	*3.39*	*3.19*	
(N):	(17):	(16):	(11):	(4):	
**IL-6**	1.02	0.89	0.87	1.06	F(2,50) = 0.74, p = 0.48
	*0.64*	*0.60*	*0.66*	*0.61*	
(N):	(20):	(20):	(14):	(7):	
**TNF-α**	1.60	1.69	1.69	1.07	F(2,48) = 0.05, p = 0.95
	*0.78*	*0.72*	*0.68*	*0.26*	
(N):	(19):	(19):	(14):	(7):	
IFN-γ *	4.07	3.17	2.08	0.92	*F(2,42) = 3.54, **p = 0.038**, η^2 ^= 0.14
	*2.37*	*1.76*	*0.96*	*0.20*	
(N):	(16):	(18):	(12):	(5):	
**IL-16**	140.23	135.18	135.90	123.92	F(2,50) = 0.08 p = 0.92
	*35.36*	*28.35*	*57.10*	*43.51*	
(N):	(19):	(21):	(14):	(7):	
**IL-10**	1.08	1.04	0.75	0.91	F(2,48) = 0.77, p = 0.47
	*0.64*	*0.75*	*0.44*	*0.66*	
(N):	(18):	(21):	(13):	(4):	
IL-13*	7.42	5.92	5.86	4.03	*F(2,31) = 2.89, **p = 0.07**, η^2 ^= 0.16
	*2.95*	*2.22*	*1.55*	*1.29*	
(N):	(14):	(11):	(10):	(3):	

The ancova covariate was age: analyses excluded sibs: see text for one-way analyses. Cytokines with proinflammatory activity include IL-1β, IL-2, IL-6, TNF-α, and IFN-γ and with antiinflammatory activity include IL-10 and IL-13. Both properties have been ascribed to IL-16 in different disorders.

IL-16 levels did not differ significantly between control and ADHD groups (F = 0.62). But across all subjects IL-16 levels correlated positively with the severity of allergy (r = +0.26, p = 0.04, n = 61). Such a relationship was also seen for the antiinflammatory IL-10 (r = +0.28, p = 0.03, n = 58). The IL-16 relationship was not significant for any group alone: but, there were indications of a possible association with oppositional and hyperactive symptoms (r = +0.22, p = 0.089/0.099) rather than the inattentive ratings implicated in the genetic study (see introduction). This association was reduced in a partial correlation controlling for allergy severity. Interestingly, there were positive and negative partial correlations respectively for IL-16 and IL-10 levels with those for S100B (r = +0.47, p = 0.07, r = -0.63, p = 0.01) in the ADHD, but not in the control groups.

The ratios of pro- to antiinflammatory interleukins provide indices of the balance that influences enzyme activity in the kynurenine metabolic pathway (hypothesis 3). For example, the ratio of proinflammatory TNF-α to antiinflammatory IL-13 (but not IL-10) showed a trend for low ADHD values (ADHD 0.27, sd 0.10; CON 0.35, sd 0.12) to increase on medication (ADHDmed 0.31, sd 0.06: F(2,29) = 3.1, p = 0.06, η^2 ^= 0.18, covariates age and gender). However, the ratios of proinflammatory IL-2 and IL-6 to antiinflammatory IL-10, IL-13 and IFN-γ did not differ between groups. In contrast, the low levels of the proinflammatory IFN-γ to antiinflammatory IL-13 ratio (but not IL-10) in the ADHDmed versus the ADHD group (CON 0.69, sd 0.21; ADHD 0.60 sd 0.19; ADHDmed 0.36, sd 0.11: F(1,21) = 11.76, p = 0.003) were attenuated with the same covariates (F(1,19) = 3.70, p = 0.07, η^2 ^= 0.16), suggesting a partial contribution of the age and gender differences. Lastly, despite relatively few data, the ratios of IL-1β to IL-10, IL-13 and IFN-γ were lower in the ADHD than the control group and increased in the ADHDmed children (F(2, 8-17) = 3.5-5.5, p = 0.05-0.02, η^2 ^= 0.29-0.54: e.g. IL-1b/IL-10, CON 0.45, sd 0.41; ADHD 0.21, sd 0.14; ADHDmed 0.72, sd 0.81).

IL-16/IL-10 ratios were of interest because of the relationship to allergic sensitivity. Compared to controls they were non-significantly lower in the ADHD and higher in the medicated group (respectively, 184 sd 140, 199 sd 141 and 265 sd 299: F(2,46) = 1.18, p = 0.32, η^2 ^= 0.05). However, the ratio correlated positively with CPRS ratings of oppositional behaviour after controlling for age (r = +0.57, p = 0.03). The correlation was reduced after control for allergic sensitivity and was absent in the ADHDmed group (r = -0.08).

**Figure 2:**
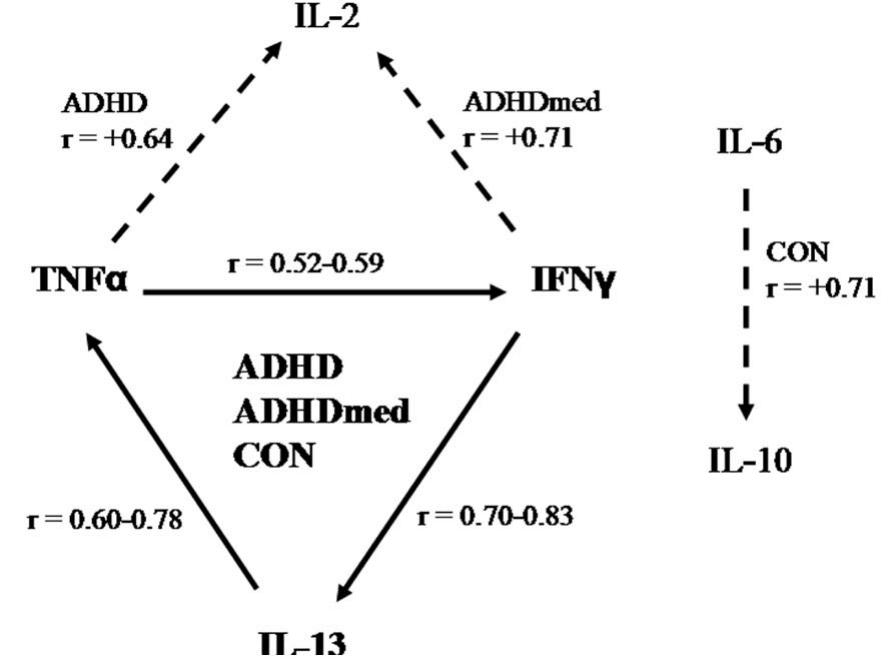
**The solid arrows illustrate the significant Pearson correlations between interleukin concentrations in ADHD, ADHDmed and Control groups of children and the broken arrows illustrate relationships specific to each group identified on the arrow**.

Figure 2 shows correlations between levels of the cytokines (p = 0.05-0.001). The figure illustrates (in bold) the associations between TNF-α, IFN-γ and IL-13 that were significant and common to each of the main groups (ADHD, ADHDmed and CON). It also shows (broken lines) that there was a further relationship unique to each group.

In summary medication tended to normalise mild alterations of cytokine levels in ADHD children. The ratio of TNF-α/IFN-γ, lower in ADHD than control children, increased in the ADHDmed group. The IL-16/IL-10 ratios reflected sensitivity to allergy and were associated with S100B levels and oppositional symptoms. While associations between some interleukins were maintained across groups (e.g. TNF-α, IFN-γ, IL-13), a potential homeostatic imbalance for others (IL-2, IL-6, IL-10) is evident from group specific relationships.

## Discussion

The principle results of this study may be summarised as follows.

### 1/S100B

a) There were no group differences for levels of S100B (ADHD ± medication):

b) In general, S100B levels were positively related to anxiety, but in ADHD children (controlling for oppositional behaviour) the relationship of lower S100B levels with anxiety was negative

c) Decreasing S100B levels in ADHD groups related to decreasing levels of IL-16, but increasing levels IL-10 (independent of age), - where IL-16 and IL-10 correlated with allergy sensitivity. IL-16 relationship to oppositional/hyperactive symptoms depended partly on allergy sensitivity.

### 2/Interleukins

a) There were no clear group difference for interleukin levels or ratios of pro/anti-inflammatory interleukins

b) Psychostimulant medication tended to normalise the slightly altered levels of pro-and anti-inflammatory interleukins and significantly increased some pro/anti-inflammatory interleukin ratios

c) Common to each group were associations between TNF-α, IFN-γ and IL-13, suggesting intact control systems. But relations of TNF-α and IFN-γ to IL-2 and IL-6 to IL-10 were specific to ADHD, ADHDmed and controls, respectively, suggesting condition/state-dependent influences in the 3 groups.

### 3/Amino acids and kynurenine metabolism

a) Tryptophan availability was moderately increased in both ADHD groups over control values.

b) A strong association of kynurenine to its toxic metabolite 3HK exclusively in controls contrasted with modest associations for neuroprotective indices in all groups

c) Medication moderated the production of 3KA in the ADHDmed group.

d) Amino acid levels and their group-specific associations with 3HK support marked catabolic activity that may indicate neural pruning activity in controls rather than ADHD children.

#### S100B

Our main hypothesis of altered serum levels of S100B in ADHD was not upheld. A trend towards lower levels of this putative biomarker of astrocyte integrity in patients with symptoms of anxiety, disturbed mood and poor self-esteem could indicate an inefficient energy supply to support active neuronal function in a subgroup of children without conduct problems. This decrease was partly predicted by the original hypothesis [6,19] and on the grounds that ADHD is very different from the severe disorders that give rise to increased S100B secretion (see introduction).

The failure of this first test of impaired astrocyte function across the whole group of ADHD children has not disproved hypothesis-1. First, synthesis of S100B is not unique to astrocytes [8]. Alterations of function relevant to neuronal support could be masked by other sources of S100B production. Second, other factors indicative of astrocyte function should be measured. One example is LIF, a cytokine released from astrocytes by the ATP associated with firing neurons, that is involved in protecting the neuronal energy supply and in the development of astrocytes and oligodendrocytes [45,46]. LIF is crucial to the development of midbrain dopaminergic (DA) cells [47] relevant to ADHD, a putatively hypodopaminergic disorder. Unfortunately the analysis here proved insensitive to LIF levels in circulation. Measures of the neuronal and glial metabolite myo-inositol (MI) should be encouraged. MI is crucial to triggering calcium release, an integral feature of glial signalling. Magneto-resonance spectroscopic (MRS) studies of infants born prematurely or at term, at risk for intrauterine insufficiency, have shown clearly that MI provides an excellent marker of the appropriate maturation of cerebral structures [48] where a delay is implicated in children with ADHD. Small MRS studies of ADHD children describe associations of MI/creatine ratios with learning, memory and language function [49] and increases of the glutamate/MI ratio that are consistent with an inadequate supply of MI [50]. Alterations are evident in several psychiatric disorders with MI decreases recorded in depressed patients [51] and increases in those with schizophrenia that correlated with increases of S100B [9].

#### Tryptophan metabolism

The neuromodulator 5-HT, for which tryptophan is a precursor, has become a major focus of interest in ADHD. Relatively speaking levels of the metabolite 5-HIAA excreted are often higher than for DA metabolites and negatively related to measures of signal detection and attention [21,23,52]. Interactions of 5-HT with the DA system in impulsivity [53], the sensitivity to expressed emotion [54] and other functional domains in ADHD have been reported [22]. The course of development and differentiation of the CNS is crucially influenced by 5-HT systems [55,56]. In the present study despite finding modestly elevated levels of tryptophan, tryptophan availability and reduced breakdown in ADHD children, we recorded no group differences for the metabolite 5-HIAA or kynurenine. Any differences of central origin may have been masked by unaltered peripheral sources. However, in each group tryptophan levels correlated with kynurenine, which in turn related to KA levels. This is consistent with the kynurenine pathway being the main metabolic route for tryptophan [20] that results in protective (KA) or potentially toxic metabolites (3HK and quinolinic acid).

KA is an NMDA antagonist mostly of glial provenance. In the nanomolar concentrations found in the mammalian brain it reduces glutamate induced excitotoxicity that often results from insult (e.g. hypoxia). As KA is also an antagonist of α7 nicotinic cholinergic binding sites [57], reduced KA levels can lead to increased mesocortical acetylcholine release. In the presence of low levels increased neostriatal and mesocortical DA release have been described [58] as being modulated by the α7 nicotinic receptor [59]. These features of low levels of KA could benefit ADHD subjects with cognitive impairment and a hypodopaminergic system. Against this background it is notable that the KA levels recorded here were quite similar between groups and were modestly correlated with other metabolic measures (amino acids).

Striking was a marked association of kynurenine with 3HK levels in the controls, that was absent in the ADHD groups. The implication of enhanced catabolic processes in controls was supported by the amino acid analysis. The levels of 6 amino acids correlated with kynurenine and 3HK exclusively in controls, whereas the ADHD group showed merely two associations with the neuroprotective index. The absence of a predominant metabolic direction in the ADHD group was not altered by medication, but became ordered to the extent that numerous amino acid levels related to kynurenine production and the tryptophan-breakdown index.

These results suggest that toxic metabolites are not influencing CNS function in young ADHD subjects, or alternatively the energy supply through NAD as a metabolite of 3HK, was available to healthy children, but restricted in ADHD [60]. The first of these two alternatives is the opposite of the prediction from hypothesis-2. We tentatively propose that the normal process of reducing and pruning neuronal processes and synapses in late childhood [61,62] was proceeding in the healthy more than in the ADHD children. This supports descriptions of delayed maturation in ADHD [63,64]. Future study should determine whether the alternative of a restricted NAD supply in ADHD is pertinent. Certainly, the absence of differences in the levels of branched-chain amino acids circulating between diagnostic groups suggests that these are not providing a supplemental source of energy for ADHD or typically developing children [36,37].

In the presence of pathology one would expect an increase in the tryptophan breakdown index (kynurenine/tryptophan) associated with an upregulation of proinflammatory IFN-γ and TNF-α that modulate the activity of the enzyme IDO [65]. Compared to controls, the present results for ADHD showed a reduced breakdown index, equivocal changes in TNF-α and only an upregulation for IFN-γ. This latter result merits attention in future study as there were decreases of IFN-γ levels and the IFN-γ/IL-13 ratios in the ADHD group on medication.

#### Cytokines

The idea to study selected interleukins came from reports of their influence on tryptophan metabolism [27]. As they can cross the blood brain barrier [66-68], immunological influences on brain function are likely and peripheral measures relevant. Pro-inflammatory interleukins influence synaptic plasticity, neurogenesis and neuromodulation [69]. Potentially relevant to ADHD, for example, is that IL-2 can modulate the development of nigrostriatal and mesolimbic DA systems [70], enhance NA utilization [71], and like IL-6 influence reaction times and working memory performance [72,73]. IL-1β is able to directly influence signalling between brain regions [74] and has been related to the development of hostility-related traits relevant to oppositionality even in healthy subjects [75]. In animal models low/high levels of IL-1β result in decreases/increases of 5-HIAA [76]. Proinflammatory interleukins (including IL-6 and TNF-α) can enhance glutamate receptor activation and inhibit GABA and glycine responses [77]. While normal physiological levels of IL-6 and TNF-α protect neurons and glia against glutamate-induced toxicity, elevated levels and toxicity can arise under conditions of hypoxia and brain damage, and are seen in a number of brain disorders [78,79]. Along with TNF-α, these proinflammatory cytokines can mediate the effects of stress on monoamine activity and cognitive performance [80,81]. Many of these effects may be mediated by neurons, but many may occur via the astrocytes, the control of energy supply and glutamate activity [82]. Antiinflammatory cytokines (e.g. IL-10) can counteract a number of these physiological and cognitive effects [83,84]. As yet there has been no study of the potential relevance of these effects to the development and expression of the features of ADHD.

Of the 8 interleukins measured, 6 showed a marginal increase in the ADHD group. IL-1β levels showed a small decrease. These measures all tended to be corrected in the ADHDmed group. These trends reached borderline significance for IFN-γ and IL-13. Hirschberg et al. [85] observed that following an insult on brain function a degree of inflammation is essential for regeneration, and in this case a small antiinflammatory response was observed. The small size of the effect may reflect its insignificance or that the levels measured were a small echo of what may have been shown earlier in development around the onset of the disorder [43]. It is worth noting that no relationship with allergy sensitivity was recorded.

The ratios of pro- to antiinflammatory cytokines did not distinguish ADHD cases from controls (hypothesis -3), and thus there is no evidence for an imbalance that would affect the induction of the enzymes (IDO and TDO). This contrasts with findings of elevated proinflammatory cytokines from adults suffering from depression [27,86] and melancholia [87]. However, low levels of the ratios of IL-1β to antiinflammatory cytokines in the ADHD group were higher in the medicated group. We would propose that the tendency of medication to 'normalise' interleukin levels does not reflect the well-known blockade of psychostimulants of the neuronal DA transporter, but rather their ability to stimulate the energy transfer functions of astrocytes [88]. This refers in particular to the uptake of circulating glucose, its transfer and utilization as lactate by neurons, or storage as glycogen in the glial cells for the supply of energy when required [89,90]. This mechanism is an important component of the hypothesis to explain variability in ADHD [6].

Lastly, the role of the pro-/antiinflammatory IL-16 cytokine is worthy of further study. In a genome wide scan of ADHD subjects [34], 2 SNPs were associated with the inattentive phenotype. While IL-16 levels did not differ between the groups studied, they correlated positively and IL-10 levels correlated negatively with the experience of allergy across all subjects: (see [91] on low IL-10 transmission reflecting paternal allergy). Exactly paralleling this were similar correlations with S100B. Indeed, within ADHD IL-16 levels correlated with ratings of oppositional behaviour. The production of IL-16 has been associated previously with asthma, and its secretion to depend on stimulation by 5-HT via 5-HT2a receptors [92]. IL-16 can also stimulate the production of IL-1β, IL-6 and TNF-α in monocytes [93]. The prevalence of allergic sensitivity has long been reported to be higher in hyperactive children [94,95] and that symptoms can be alleviated by removal of allergens [96]. Indeed, allergies may be over twice as frequent among ADHD children with internalizing problems as healthy children, or those with ADHD and comorbid conduct disorder [97]. This supports the association with S100B described above.

#### Limitations

The principle limitations in this study relate to the small number of subjects in each group, that in turn limits the power of the analyses. The number of sibling controls volunteering to participate was also smaller than anticipated. It was necessary to control for an imbalance in gender and age in the analyses. This arose through the difficulty of persuading children to provide samples for research rather than clinical reasons. Technical difficulties in the biochemical analyses further reduced the number of data for some measures. However, the study was intentionally preliminary and the aims entirely exploratory without conservative correction, to see if there were any indications of alterations in the levels of potentially relevant cytokines that merited a follow-up with an intensive study. Further, an interpretation of the effects of medication is limited by the cross-sectional between-group design of the study. It would be preferable for a future in-depth study to employ a longitudinal within-subject design.

## Conclusions

Over the last 10 years it has become recognized that inflammation may represent a common mechanism of neuropsychiatric disorders as well as somatic disease (e.g. depression, schizophrenia, and dementia). In the brain this can be represented by excitotoxicity and impairment of glial function. We show here that one marker of glial function (S100B) is not dysfunctional across the major manifestations of ADHD, even though its activity may be low in those with internalizing comorbidities. We also show that there is no major dysfunction in the balance of pro- and anti-inflammatory interleukins that modulate the production of toxic and protective kynurenine-related metabolites of tryptophan. However, the results do show a certain disorder of catabolism that together may influence features of ADHD (e.g. oppositional characteristics). Nonetheless, there were two stronger findings. The first is that toxic kynurenine metabolites are a feature of late healthy childhood and this may point to the nature of the mechanism of pruning of neural elements widely reported in normal development. The second is that there is a normalising influence of psychostimulant medication on immunological characteristics that points to a widely neglected locus of pharmacotherapeutic action, namely glial function. This parallels previously documented actions of antidepressant therapy [98].

## Abbreviations

ADHD: Attention-deficit/hyperactivity disorder; BMI: Body mass index; CON: Control; DA: Dopamine; DSM-IV: Diagnostic and Statistical Manual of the American Psychiatric Association, 4^th ^edition; 5-HIAA: 5-hydroxy-indole-acetic acid; HPLC: High Performance Liquid Chromatography; 5-HT: Serotonin; IL: Interleukin; IFN-γ: Interferon gamma; KA: Kynurenic acid; 3HK: 3-hydroxy-kynurenine; LIF: Leukemia inhibitory factor; MRS: Magneto-resonance spectroscopy; PACS: Parental Account of Children's Symptoms; RT: Response time; SES: Socio-economic scale; Sibs: Siblings; SD: Standard Deviation; TNF-α: Tumour necrosis factor alpha

## Competing interests

RDO, MD and BGS declare that they have no competing interests beyond the source of finance for the study (see below). AMM & MJS have patented the use of tryptophan pathway metabolites as neurodegenerative markers for depression and related psychiatric diseases

## Authors' contributions

RDO was involved in conceiving and organizing the study as well as analysing the data and writing the report. The organization was refined by BGS who with MD was involved in recruitment, diagnosis and logistics. MJS and AMM advised on the biochemical items for study, organized and ran the biochemical analyses and contributed to the strategy for the study and report. The ideas and hypotheses derived from discussion of the work of AMM and RDO. All authors contributed to and approved the final manuscript.

## Authors' information

The study design was intentionally exploratory and the interpretation accordingly conditional. The presentation is aimed at informing both the disciplines of ADHD (psychiatric) and psychoimmunology (basic research) for whom certain elements may be more or less familiar and include negative and positive findings that may both guide future investigations. Some of these data were communicated at the 10^th ^Psychoimmunology Expert meeting at Ulm/Günzburg, 12-14^th ^Nov. 2009 ([99]).
